# Different Degrees of Sulfated Laminaria Polysaccharides Recovered Damaged HK-2 Cells and Inhibited Adhesion of Nano-COM and Nano-COD Crystals

**DOI:** 10.1155/2024/8843214

**Published:** 2024-01-02

**Authors:** Qiu-Shi Xu, Zhi-Jian Wu, Jian-Ming Sun, Jing-Hong Liu, Wei-Bo Huang, Jian-Ming Ouyang

**Affiliations:** ^1^Department of Urology, The First People's Hospital of Chenzhou, Hunan, Chenzhou 423000, China; ^2^Institute of Biomineralization and Lithiasis Research, Jinan University, Guangzhou 510632, China

## Abstract

**Purpose:**

The crystal adhesion caused by the damage of renal tubular epithelial cells (HK-2) is the key to the formation of kidney stones. However, no effective preventive drug has been found. This study aims to explore the recovery effects of four Laminaria polysaccharides (SLPs) with different sulfate (–OSO_3_^–^) contents on damaged HK-2 cells and the difference in the adhesion of damaged cells to nanometer calcium oxalate monohydrate (COM) and calcium oxalate dihydrate (COD) before and after recovery.

**Methods:**

Sodium oxalate (2.6 mmol/L) was used to damage HK-2 cells to establish a damaged model. SLPs (LP0, SLP1, SLP2, and SLP3) with –OSO_3_^–^ contents of 0.73%, 15.1%, 22.8%, and 31.3%, respectively, were used to restore the damaged cells, and the effects of SLPs on the adhesion of COM and COD, with a size of about 100 nm before and after recovery, were measured.

**Results:**

The following results were observed after SLPs recovered the damaged HK-2 cells: increased cell viability, restored cell morphology, decreased reactive oxygen levels, increased mitochondrial membrane potential, decreased phosphatidylserine eversion ratio, increased cell migration ability, reduced expression of annexin A1, transmembrane protein, and heat shock protein 90 on the cell surface, and reduced adhesion amount of cells to COM and COD. Under the same conditions, the adhesion ability of cells to COD crystals was weaker than that to COM crystals.

**Conclusions:**

As the sulfate content in SLPs increases, the ability of SLPs to recover damaged HK-2 cells and inhibit crystal adhesion increases. SLP3 with high –OSO_3_^–^ content may be a potential drug to prevent kidney stones.

## 1. Introduction

The main inorganic component of kidney stones is calcium oxalate (CaOx), including CaOx monohydrate (COM) and dihydrate (COD) [1, 2]. Various chronic diseases, such as chronic kidney disease and diabetes, could lead to the formation of CaOx kidney stones [3, 4].

The oxidative damage to renal tubular epithelial cells (HK-2) caused by reactive oxygen species (ROS) is the main reason for the formation of CaOx stones [5]. ROS is a normal metabolite in cells and tissues, and its level is kept in balance by the antioxidant effect of many substances, including superoxide dismutase, glutathione peroxidase, and catalase [6]. When ROS is overproduced, cells are damaged [7].

HK-2 cells damaged by oxalate have stronger adhesion to CaOx crystals than normal HK-2 cells, which may be related to the overexpression of annexin A1 (ANXA1) and transmembrane protein (CD44) on the surface of damaged cells [8–10]. Zhang et al. [8] have found that the surface of damaged HK-2 cells could express negatively charged crystal adhesion molecules, such as osteopontin and CD44, thereby further promoting the adhesion and aggregation of crystals and increasing the risk of kidney stone formation. Studies have shown that the COM crystal-binding proteins on the apical membrane of HK-2 cells (such as ANXA1, *α*-enolase, and HSP90) act as potential crystal receptors for the adhesion of COM crystals on the cell apical surface and the retention of COM crystals in the tube. Moreover, the subsequent formation of stones is crucial [9].

Polysaccharides could recover damaged cells [11–13]. Li et al. [11] used four tea polysaccharides with different molecular weights to recover HK-2 cells after oxalate damage. The results show that the recovered cell viability and the mitochondrial membrane potential (ΔΨ_*m*_) increased, whereas the ROS levels and lactate dehydrogenase release decreased. These phenomena reduced the adhesion of crystals on the cell surface, increased the amount of CaOx crystals endocytosed by cells, and helped inhibit the formation of kidney stones. Liu et al. [12] have found that *Lycium barbarum* polysaccharide could eliminate oxidation products 8-hydroxydeoxyguanosine and lipid peroxide and increase the anti-Müllerian hormone expression to recover ovarian damage. *Hizikia fusiforme* polysaccharides could remarkably reduce the cytotoxicity, the level of intracellular ROS, and apoptosis induced by hydrogen peroxide in Vero cells [13]. In addition, *Hizikia fusiforme* polysaccharides have a strong protective effect on the oxidative stress induced by hydrogen peroxide in zebrafish.

The functional groups in polysaccharides (such as sulfate and carboxyl) are important factors that affect the biological activity of polysaccharides [14]. For example, Chen and Huang [15] have sulfated cushaw polysaccharides to obtain SP1 and SP2 with substitution degrees of 0.28 and 0.53, respectively. Their scavenging abilities to the superoxide anion follow the order SP2 > SP1 > polysaccharide, indicating that increasing the sulfation degree of polysaccharide results in stronger antioxidant activity. Han et al. [16] modified *Cyclocarya paliurus* polysaccharide (CPP) by sulfation to obtain the sulfated derivative S–CPP. The results showed that S–CPP exhibited stronger immunomodulatory activity than CPP. Carboxymethylated cushaw polysaccharide also demonstrated a better ability to scavenge superoxide anions and hydroxyl radicals than the original cushaw polysaccharide [17].

As a traditional medicine and marine vegetable, *Laminaria japonica* has a history of more than 1000 years. *L. japonica* is distributed in circumlittoral countries and has high nutritional value and healthcare functions [18]. The *Laminaria* polysaccharide (LP) is the main active ingredient and has a wide range of pharmacological properties, such as anti-inflammatory, antioxidant, and antiatherosclerotic properties [19, 20]. Abdel-Daim et al. [21] have found that the fucoidan extracted from *L. japonica* could improve the oxidative stress, inflammation, and damage to the rat cardiac, hepatic, and renal tissues induced by diazinon. Long et al. [22] have studied the effect of purified *L. japonica* polysaccharide (LJP61A) on the renal function of a mouse model of adenine-induced chronic renal failure (CRF). The results showed that the LJP61A could reduce the serum creatinine and the blood urea nitrogen of mice, improve the urine biochemical index, and regulate the electrolyte disturbance of CRF mice, suggesting that it could improve the renal function of mice with adenine-induced CRF.

In our early work, we have sulfated the original laminaria polysaccharide (LP0, with sulfate contents of 0.73%), and three sulfated polysaccharides with –OSO_3_^–^ content of 15.1% (SLP1), 22.8% (SLP2), and 31.3% (SLP3) were obtained [23]. The antioxidant activity of these SLPs and their ability to regulate CaOx crystal growth were investigated. The results showed that SLPs with high –OSO_3_^–^ content had stronger activity. In this paper, the recovery effect of the four SLPs on the damaged HK-2 cells and the difference in cell adhesion to nanosized COM and COD crystals before and after the recovery were explored to further study the preventive and treatment effects of polysaccharides. We hope to provide inspiration for finding new antistone drugs.

## 2. Materials and Methods

### 2.1. Materials and Apparatus

**Figure 1:**
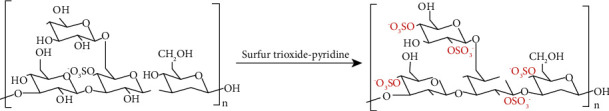
LP0 sulfation reaction equation.

The polysaccharides with sulfate contents of 0.73% (LP0), 15.1% (SLP1), 22.8% (SLP2), and 31.3% (SLP3) were obtained according to reference [23], and the sulfation reaction equation of LP0 was shown in Figure 1. DMEM/F-12 culture medium and fetal bovine serum were purchased from Gibco (USA). A cell counting kit (CCK-8) was purchased from Dojindo Laboratory (Kumamoto, Japan). 3-Aminopropyl triethoxysilane (APTES) and fluorescein isothiocyanate (FITC) were purchased by Macklin Biochemical Technology (Shanghai, China). 2′,7′-dichlorodihydrofluorescein diacetate (DCFH-DA), 4,6-diamidyl-2-phenylindole (DAPI), 1,1′-dioctadecyl-3,3,3′,3′-tetramethylindocarbocyanine perchlorate (DiI), and 5,5′,6,6′-tetrachloro-1,1′, 3,3′-tetraethylbenzimi-dazolylcarbocyanine iodide (JC-1) dyes were purchased from Shanghai Beyotime Bio-Tech Co., Ltd. (Shanghai, China). HK-2 cells were purchased from the Shanghai Cell Bank of the Chinese Academy of Sciences (Shanghai, China). Oxalate and other chemical reagents were all analytically pure and purchased from Guangzhou Chemical Reagent Factory of China (Guangzhou, China).

The following apparatus was used in this study: multifunctional microplate reader (SafireZ, Tecan, Switzerland), flow cytometer (FACS Aria, BD, USA), X-L type environmental scanning electron microscope (Phillps, Eindhoven, Netherlands), inverted fluorescence microscope (Olympus, U-HGLGPS, Japan), confocal laser scanning microscope (LSM510 META DUO SCAN, Zeiss, Germany), and optical microscope (Olympus, TH4-200, Japan).

### 2.2. Cell Culture and Groupings

HK-2 cells were cultured in DMEM/F-12 medium containing 10% fetal bovine serum at 37°C and 5% CO_2_ saturated humidity. The cells were passaged by trypsin digestion. When they reached 80%–90% confluence, they were washed twice with PBS buffer. Then, 0.25% trypsin-EDTA digestive solution was added, and the cells were placed in an incubator at 37°C for 5 min. The degree of digestion was observed under the optical microscope. After moderate digestion, DMEM/F-12 cell culture medium containing 10% fetal bovine serum was added to terminate the digestion, and the cells were fully dispersed to form a single-cell suspension.

The above single-cell suspension with a concentration of 1.0 × 10^5^ cells/mL was inoculated into 6-, 12-, and 96-well culture plates at a volume of 2 mL/well, 1 mL/well, and 100 *μ*L/well, respectively. After 24 h of incubation, the culture medium was removed, and the cells were washed twice with PBS. The cells were divided into three groups: (A) normal group, where only serum-free culture medium was added; (B) damage group, where serum-free medium containing 2.6 mmol/L oxalate was added and incubated for 3.5 h; (C) polysaccharide recovery group, wherein after 3.5 h of Na_2_Ox damage, SLPs with different sulfate contents (0.73%, 15.1%, 22.8%, and 31.3%) at a concentration of 80 *μ*g/mL were added, and the cells were recovered for 12 h.

### 2.3. The Phenomenon Observation of Polysaccharide Repairing Damaged HK-2 Cells

#### 2.3.1. Cell Viability Detection and Cell Morphology Observation

The cell seed plates were the same as those described in Section 2.2. The cells were divided into four groups: (A) cell-free culture medium group (background control group); (B) normal group, where only serum-free culture medium was added; (C) damage group, where serum-free medium containing 2.6 mmol/L oxalate was added and incubated for 3.5 h; (D) polysaccharide recovery group, wherein after 3.5 h of Na_2_Ox damage, SLPs with different concentrations of 20, 40, and 80 *μ*g/mL were added to recover the damaged cells for 12 h. Then, 10 *μ*L of CCK-8 reagent was added to each well and incubated in an incubator at 37°C for 2 h. The absorbance (*A*) was measured at 450 nm by a microplate reader, and the cells under each condition were measured in parallel for 5 wells. The average value of A was calculated to detect the recoverability of polysaccharides as follows: cell viability (%) = *A* (treatment group)/*A* (control group) × 100.

The experimental groups were the same as those in Section 2.2. After the action time was reached, the cell morphology in the 6-well plate was observed under the optical microscope.

#### 2.3.2. Cell Healing Ability Assay

The cell seed plate was the same as that described in Section 2.2. When the action time reached, the supernatant was removed, and a sterile 200 *μ*L gunhead was used to draw lines in a sample culture dish in a certain direction. After the cells were washed twice with PBS, a fresh culture medium was added to continue the culture. At intervals, the changes in scratch spacing were observed under the optical microscope, photos were taken, and the cell healing rate was calculated.

### 2.4. Detection of Cell Damage-Related Indexes

#### 2.4.1. ROS Level Detection

The experimental groups were the same as those in Section 2.2. When the action time reached, the supernatant in the 6-well plate was removed. Subsequently, 1 mL DCFH-DA diluent was added to each well, which was then incubated in the incubator at 37°C for 30 min in the dark. The serum-free medium was washed three times. The 6-well plate was observed by the inverted fluorescence microscope. Finally, ImageJ software was used for fluorescence semiquantitative analysis of ROS.

#### 2.4.2. Mitochondrial Membrane Potential (ΔΨ_*m*_) Detection

The experimental groups were the same as those in Section 2.2. When the action time reached, the supernatant in the 6-well plate was removed. Next, 1 mL of JC-1 working solution with a concentration of 20 *μ*mol/L was added to each well, which was then incubated at 37°C for 30 min in the dark. Afterwards, the cells were washed twice with PBS to remove excess JC-1 and observed by the inverted fluorescence microscope. Finally, ImageJ software was used for fluorescence semiquantitative analysis of JC-1.

#### 2.4.3. Detection of Phosphatidylserine (PS) Eversion Ratio

The experimental groups were the same as those in Section 2.2. When the action time reached, the supernatant was removed, and the cells were washed twice with PBS. Then, 100 *μ*L of binding buffer and 10 *μ*L of FITC-labeled Annexin-V (20 *μ*g/mL) were added and incubated at room temperature for 30 min in the dark. These samples were quantitatively detected by the flow cytometer.

### 2.5. Detection of Crystal Adhesion to Cells

#### 2.5.1. Detection of ANXA1, CD44, and HSP90 Expression Levels

The experimental groups were the same as those in Section 2.2. When the action time reached, the cells were washed twice with pre-cooled PBS and drained. Then, the cells were lysed with 120 *μ*L RIPA lysis buffer, and the cells and reagents were scraped with a cell scraper. The cells were collected into 1.5 mL EP tubes and then ultrasonically lysed three times at 20 s each time, with an interval of 5 s. Next, they were centrifuged at 4°C and 13000 rpm for 20 min. The supernatant was collected, and the protein concentration of all samples was balanced. These samples were added to a 12% tris-glycine SDS-polyacrylamide gel, and the gel was run for 2 h at 120 V. Subsequently, the protein was transferred to a polyvinylidene fluoride membrane. The membrane was blocked with 5% skimmed milk for 1 h, soaked with ANXA1 and CD44 primary antibody at 4°C overnight, and then washed three times with TBST on a shaker at room temperature for 10 min each time. The secondary antibody was diluted 10,000 times with TBST and incubated in the dark for 2 h, washed three times with TBST, and then developed.

#### 2.5.2. SEM Observation of Crystal Adhesion

The cell seed plates were the same as those in Section 2.2. The cells were transferred to 4°C for 0.5 h to inhibit cell endocytosis of crystals [24]. The experimental models were divided into three groups: (A) normal group, where normal cells were added with freshly prepared nano-COM or nano-COD (size of about 100 nm); (B) damage group, where nano-COM or nano-COD was added after 3.5 h of Na_2_Ox injury; (C) polysaccharide recovery group, wherein nano-COM or nano-COD was added after LP0 and SLP3 polysaccharides recovered the damaged cells for 12 h. The crystal concentration was 200 *μ*g/mL. The adhesion time was 1 h, and the cells were cultured at 4°C. After the culture time was reached, the cells were washed twice with cold PBS and fixed with 2.5% glutaraldehyde at 4°C for 24 h. Then, they were dehydrated with gradient ethanol (30%, 50%, 70%, 90%, and 100% separately), dried under the critical point of CO_2_, and sprayed with gold. The crystal adhesion was observed by SEM referred to our previous method [25], and the measurement parameters are acceleration voltage (EHT, extra high tension): 5.00 kV; magnification (Mag): 4.0 KX; secondary electron detector (Signal *A* = SE2); working distance (WD): 5.3∼6.2 mm.

#### 2.5.3. Fluorescent Labeling of Nano-COM and COD Crystals

In accordance with reference [26], FITC-labeled nano-COM and nano-COD were prepared by a two-step reaction. APTES (5 mL) was mixed with 0.05 g COM or COD in 50 mL anhydrous ethanol at 74°C for 3 h. Subsequently, 0.025 g FITC was added, and the reaction was maintained for 6 h. Free FITC and APTES were removed using a dialysis bag (Mw 8000–14000). Green fluorescently labeled FITC-COM or FITC-COD particles were obtained after drying. A certain amount of nano-COM and nano-COD crystals before and after fluorescence labeling was resuspended in anhydrous ethanol separately. Ultrasonication was carried out for 10 min, and then, they were added to a 6-well plate and observed under the inverted fluorescence microscope. A total of 10,000 crystals were detected by flow cytometry, and the percentage of labeled crystals was statistically analyzed.

#### 2.5.4. Confocal Qualitative Observation of Cell Adhesion

The experimental groups were the same as in Section 2.2. After 12 h of restoration, the confocal dishes were transferred into a 4°C environment and cultured for 0.5 h to inhibit the endocytosis of the crystals. Then, 200 *μ*g/mL of freshly prepared FITC-COM or FITC-COD was added and cultured at 4°C for 1 h. After the cells were washed twice with cold PBS, the cell membrane was stained with DiI for 20 min [27], and the nucleus was stained with DAPI for 10 min [28], fixed with paraformaldehyde (4%) for 10 min, and washed three times with PBS. Finally, the samples were observed under the confocal laser scanning microscope.

#### 2.5.5. Quantitative Detection of Cell Proportion with Adhered Crystals

The cell seed plates were the same as those in Section 2.2. The experimental models were divided into three groups: (A) normal group, where normal cells were added with freshly prepared 200 *μ*g/mL FITC-COM or FITC-COD crystals; (B) damage group, where FITC-COM or FITC-COD crystals were added after Na_2_Ox injury for 3.5 h; (C) polysaccharide recovery group: whereas FITC-COM or FITC-COD crystals were added after LP0 and SLP3 polysaccharides recovered the damaged cells for 12 h. All three groups of cells were first transferred to 4°C and cultured for 0.5 h to inhibit the endocytosis of crystals. After FITC-COM or FITC-COD was added, the cells underwent adhesion for 1 h and cultured at 4°C. The culture medium was then removed, and the cells were washed twice with cold PBS to remove the unadherent and loosely adhered crystals. After trypsin digestion was performed, the cells were resuspended in PBS, and the proportion of cells adhering to the crystals was detected by the flow cytometer. The cells with a FITC signal could be regarded as cells with adherent crystals.

### 2.6. Statistical Analysis

The experimental results were statistically analyzed by IBM SPSS Statistics 26 software. Data were expressed as the mean ± SD. One-way analysis of variance was used for comparison among multiple groups, followed by the Tukey test. ^*∗*^*P* < 0.05, ^*∗∗*^*P* < 0.01, and *P* > 0.05 indicated a significant difference, extremely significant difference, and no significant difference, respectively.

## 3. Results

### 3.1. SLPs Can Repair Damaged HK-2 Cells: Observation

#### 3.1.1. Increased Vitality of Damaged HK-2 Cells after SLP Recovery

**Figure 2:**
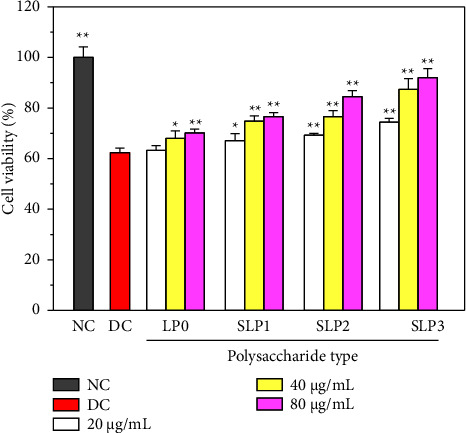
The CCK-8 method was used to detect the cell viability before and after the recovery of damaged HK-2 cells by SLPs at different concentrations. Oxalate damage concentration: 2.6 mmol/L; damage time: 3.5 h; polysaccharide concentration: 20, 40, and 80 *μ*g/mL; recovery time: 12 h; NC: normal control; DC: damaged control. Compared with the DC group, ^*∗*^*P* < 0.05, ^*∗∗*^*P* < 0.01.

As shown in Figure 2, after the HK-2 cells were damaged by 2.6 mmol/L oxalate for 3.5 h, the cell viability decreased from 100.00% ± 4.16% to 62.32% ± 1.85%, indicating that oxalate caused evident damage to cells.

When four polysaccharides at different concentrations (20–80 *μ*g/mL) were used to recover the damaged cells, the cell viability improved in a concentration-dependent manner, and the recovery effect was best at 80 *μ*g/mL. For example, after recovery using SLP3 at concentrations of 20, 40, and 80 *μ*g/mL, the cell viability improved from 62.32% ± 1.85% to 74.42% ± 1.47%, 87.39% ± 4.21%, and 92.00% ± 3.64% in the damaged group, respectively.

The recovery effect of SLPs on the HK-2 cells was positively correlated with the sulfate content in polysaccharides. For example, after 80 *μ*g/mL LP0, SLP1, SLP2, and SLP3 were used for recovery, the cell viabilities were 70.14% ± 1.54%, 76.56% ± 1.62%, 84.48% ± 2.37%, and 92.00% ± 3.64%, respectively. SLP3, which had the highest sulfate content, demonstrated the best recovery effect.

#### 3.1.2. Restored Cell Morphology after SLP Recovery

**Figure 3:**
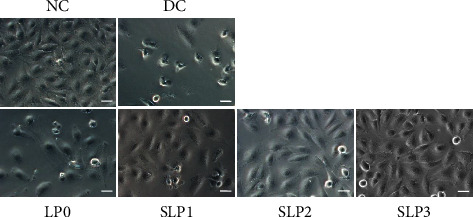
The cell morphology was observed by optical microscope before and after SLPs recovered damaged HK-2 cells. Oxalate damage concentration: 2.6 mmol/L; damage time: 3.5 h; polysaccharide concentration: 80 *μ*g/mL; recovery time: 12 h; scale bars: 50 *μ*m; NC: normal control; DC: damaged control.

Under an optical microscope (Figure 3), the cells in the normal group were evenly distributed. Cell morphology was full, and cells were closely connected. However, a remarkably reduced number of cells, loose cell distribution, destroyed connection between cells, and shrunken cell morphology were observed in the damage group. After being restored by SLPs, the degree of cell damage was reduced. The number of cells in the recovery group was remarkably more than that in the damage group. The connection between cells was gradually restored, and the cell morphology gradually recovered. A high sulfate content of polysaccharides resulted in a strong ability to restore the damage. Among them, the cell morphology after SLP3 recovery was closest to that of the normal group.

#### 3.1.3. Restored Cell Healing Ability after SLP Recovery

**Figure 4:**
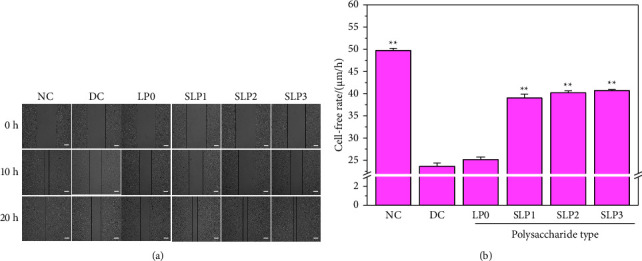
Effects of SLPs on cell healing before and after recovery of damaged HK-2 cells. (a) Cell healing observed by optical microscope. It can be seen that the cell scratch healing rate of SLP1∼SLP3 repair groups is significantly increased than that of the injury group. (b) Statistical results of the cell-free width within 24 h oxalate damage concentration: 2.6 mmol/L; damage time: 3.5 h; polysaccharide concentration: 80 *μ*g/mL; recovery time: 12 h; scale bars: 500 *μ*m. NC: normal control; DC: damaged control. Compared with the DC group, ^*∗*^*P* < 0.05, ^*∗∗*^*P* < 0.01.

As shown in Figure 4(a), the cells in the normal group were cultured for 24 h, and the number of cells in the scratches grew rapidly to close to healing, and the healing rate was the fastest (49.73 ± 0.48 *μ*m · h^−1^) (Table 1). By contrast, the wound healing rate of cells in the damage group was the slowest (23.65 ± 0.70 *μ*m · h^−1^). After the cells in the damage group were recovered for 12 h by SLPs, the number of cells and the healing rate of scratches improved to varying degrees (25.15–40.70 *μ*m · h^−1^) (Figure 4(b)), and SLP3 was the most conducive to the healing of scratches.

### 3.2. SLPs Can Repair Damaged HK-2 Cells: Biochemical Indicators

#### 3.2.1. Decreased ROS Levels of Cells after SLP Recovery

**Figure 5:**
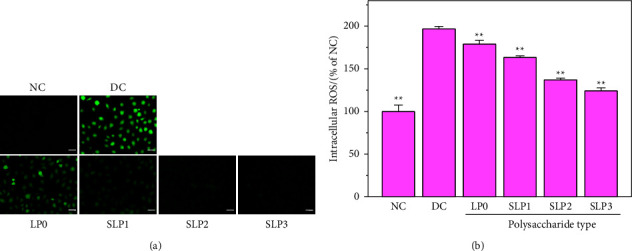
ROS levels were observed by fluorescence microscope before and after SLPs recovered damaged HK-2 cells. (a) Fluorescence microscope images. (b) Statistical results of ROS fluorescence intensity. Oxalate damage concentration: 2.6 mmol/L; damage time: 3.5 h; polysaccharide concentration: 80 *μ*g/mL; recovery time: 12 h; scale bars: 50 *μ*m. NC: normal control; DC: damaged control. Compared with the DC group, ^*∗*^*P* < 0.05, ^*∗∗*^*P* < 0.01.

The fluorescence microscopy showed that the ROS fluorescence intensity of the cells in the damage group was significantly higher than that of the cells in the normal control group (*p* < 0.01) (Figure 5(a)). After recovered by SLPs, the ROS fluorescence intensity was significantly reduced, and increasing the sulfate content in polysaccharides gradually decreased the ROS fluorescence intensity (Figure 5(b)), indicating that SLP3 had the best recovery effect.

#### 3.2.2. Increased ΔΨ_*m*_ after SLP Recovery

**Figure 6:**
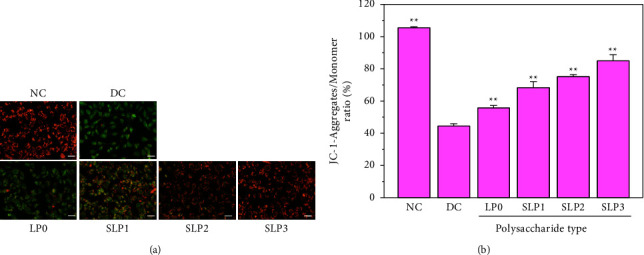
Mitochondrial membrane potential was observed by fluorescence microscope before and after SLPs recovered damaged HK-2 cells. (a) Fluorescence microscope images. (b) Statistical results of JC-1 fluorescence intensity. Oxalate damage concentration: 2.6 mmol/L; damage time: 3.5 h; polysaccharide concentration: 80 *μ*g/mL; recovery time: 12 h; scale bars: 50 *μ*m. NC: normal control, DC: damaged control. Compared with the DC group, ^*∗*^*P* < 0.05, ^*∗∗*^*P* < 0.01.

JC-1 is widely used in the detection of ΔΨ_*m*_ [29]. When ΔΨ_*m*_ is high, JC-1 could realize change from green fluorescence to orange fluorescence. As shown in Figure 6, the orange fluorescence of the cells in the normal group was the most evident, whereas the cells in the damage group showed significant green fluorescence, indicating that the ΔΨ_*m*_ was greatly reduced. After the cells in the damage group were recovered by SLPs, the orange fluorescence was enhanced to varying degrees, and the orange fluorescence of the SLP3 recovery group was the closest to that of the normal group. This finding showed that SLPs had a significant recovery effect on cells, and the recovery effect was positively correlated with the sulfate content of polysaccharides (Figure 6(b)).

#### 3.2.3. Decreased PS Eversion Ratio after SLP Recovery

**Figure 7:**
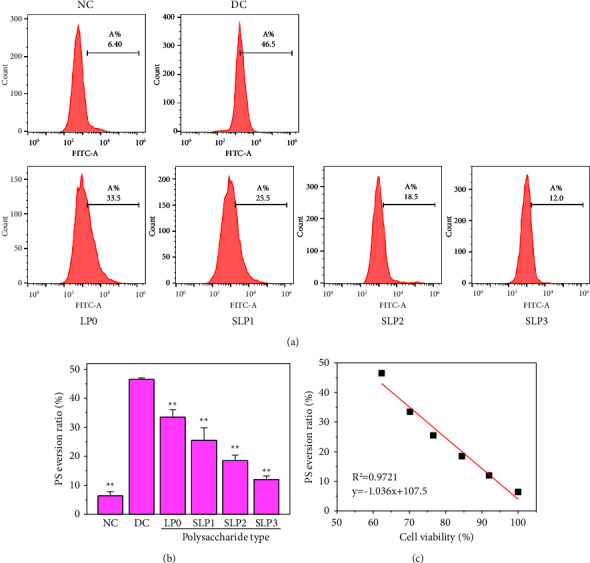
Detection of phosphatidylserine (PS) eversion ratio. (a) Flow cytometry was used to detect the PS eversion ratio of HK-2 cells. (b) Statistical results of PS eversion ratio. (c) Relationship between PS eversion amount on the cell surface and cell viability. Oxalate damage concentration: 2.6 mmol/L; damage time: 3.5 h; polysaccharide concentration: 80 *μ*g/mL; recovery time: 12 h; NC: normal control, DC: damaged control. Compared with the DC group, ^*∗*^*P* < 0.05, ^*∗∗*^*P* < 0.01.

The PS eversion ratio of cells before and after SLP recovery was detected using flow cytometry (Figure 7). The PS eversion ratio of cells in the normal group was 6.40%, whereas that of cells in the damage group increased to 46.5%. After the damaged cells were recovered by SLPs, the PS eversion ratio decreased to varying degrees, i.e., 33.5% (LP0), 25.5% (SLP1), 18.5% (SLP2), and 12.0% (SLP3). Therefore, a high sulfate content of polysaccharides was conducive to the reduction in the eversion ratio of PS.

### 3.3. SLPs Can Inhibit Crystal Adhesion to Cells

#### 3.3.1. Decreased Expression Levels of ANXA1 and CD44 after SLP Recovery

**Figure 8:**
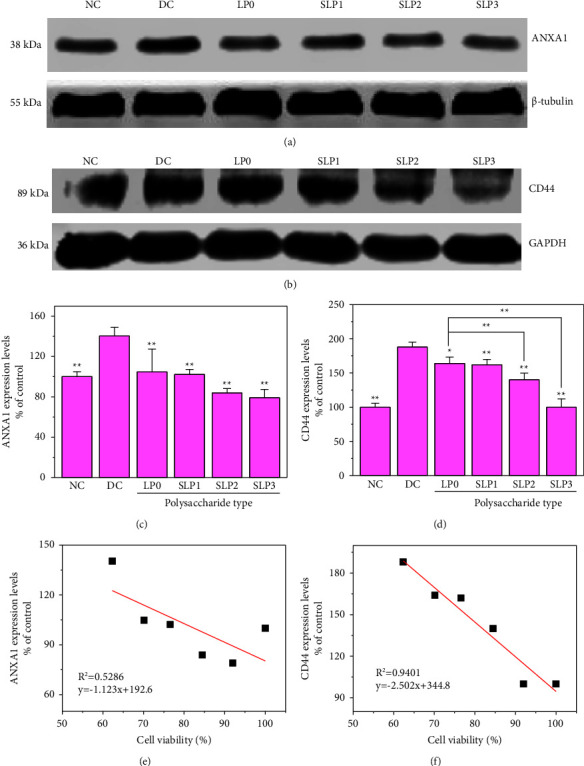
Western blot was used to detect the expression levels of annexin A1 (ANXA1) and transmembrane protein (CD44). (a) Western blot of ANXA1. (b) Western blot of CD44. (c, d) Statistical results of ANXA1 and CD44 relative expression levels. (e, f) Relationship between cell viability and the expression levels of ANXA1 and CD44 on the cell surface. Oxalate damage concentration: 2.6 mmol/L; damage time: 3.5 h; polysaccharide concentration: 80 *μ*g/mL; recovery time: 12 h; NC: normal control; DC: damaged control. Compared with the DC group, ^*∗*^*P* < 0.05, ^*∗∗*^*P* < 0.01.

Figure 8 shows the expression differences of ANXA1 and CD44 before and after SLPs recovered the damaged HK-2 cells. The expression levels of ANXA1 and CD44 in the damage group were significantly higher than those in the normal group (*p* < 0.01), indicating that oxalate damage promoted the occurrence of adhesion. After the damaged cells were recovered by SLPs, the expression levels of ANXA1 and CD44 showed different degrees of reduction. The expression levels of ANXA1 and CD44 were negatively correlated with the sulfate content of polysaccharides. In particular, increased sulfate content in SLPs decreased the expression levels of ANXA1 (Figure 8(c)) and CD44 (Figure 8(d)).

#### 3.3.2. Fluorescence Labeling of CaOx Crystals

**Figure 9:**
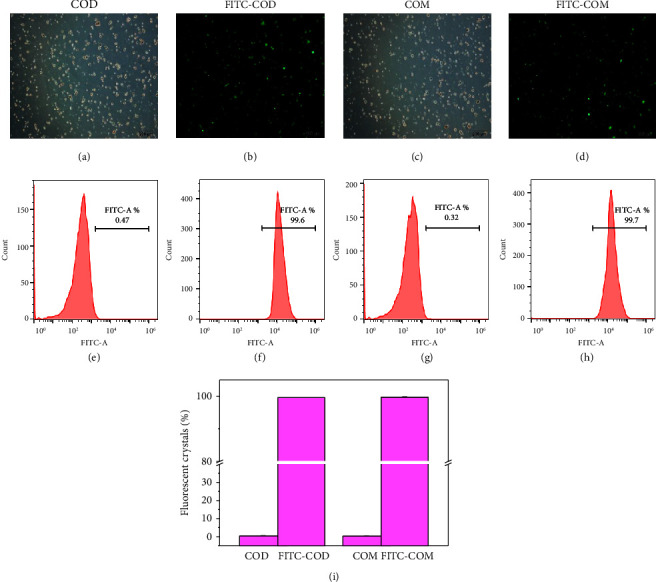
Fluorescence labeling of calcium oxalate crystals. Images of COD crystals (a, b) and COM crystals (c, d) before and after FITC fluorescence labeling. Flow cytometry analysis of COD (e, f) and COM crystals (g, h) before and after FITC fluorescence labeling. Statistical results of COD and COM crystals before and after FITC fluorescence labeling (i). FITC: fluorescein isothiocyanate.

FITC was used for the fluorescent labeling of CaOx (COD and COM) crystals. After being labeled, the crystals showed green fluorescence (Figure 9). The morphology of the two crystals was unchanged before and after the fluorescent labeling. In addition, flow cytometry tests were performed on the COD and COM crystals before and after the fluorescent labeling, and the results showed that the fluorescently labeled COD and COM crystals were as high as 99.6% and 99.7%, respectively. Therefore, fluorescence-labeled crystals could be used for the subsequent adhesion experiments. The cells that showed green fluorescence under the fluorescence microscope or FITC signals in the flow cytometer could be considered to have adhered to the crystals.

#### 3.3.3. Qualitative Observation of Crystal Adhesion on Cell Surface

**Figure 10:**
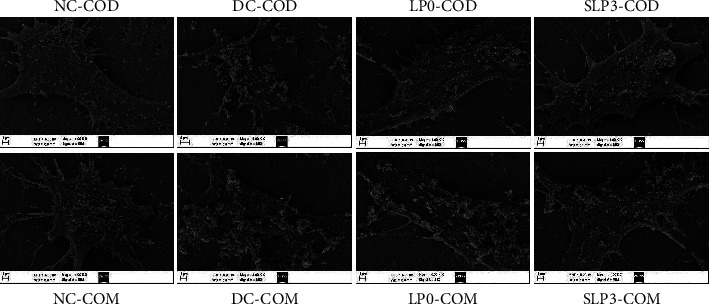
Differences in the adhesion of nano-COM and nano-COD crystals before and after the recovery of damaged HK-2 cells by LP0 and SLP3. Oxalate damage concentration: 2.6 mmol/L; damage time: 3.5 h; polysaccharide concentration: 80 *μ*g/mL; recovery time: 12 h nano-COM and nano-COD concentration: 200 *μ*g/mL; adhesion time: 1 h NC: normal control, DC: damaged control.

The adhesion differences in nano-COM and nano-COD crystals on the cell surface before and after SLPs recovered the damaged HK-2 cells were observed and compared using SEM. As shown in Figure 10, for the nano-COM crystals, the cells in the normal group had a small amount of adhesion to the crystals, whereas those in the damage group had considerable adhesion to the crystals. After LP0 and SLP3 were used for recovery for 12 h, the adhesion of cells to the crystals showed different degrees of reduction, and the SLP3 recovery group had less adhesion to the crystals than the LP0 recover group. This result indicated that SLPs had a recovery effect on HK-2 cells, and SLP3 with high sulfate content had a good recovery effect. Similar to nano-COM, the cell adhesion to nano-COD followed the order: damage group > LP0 > SLP3 > normal group. The experiment also showed that for the same group of cells (such as the damage or recovery group), the adhesion of cells to COM crystals was greater than that to COD crystals.

#### 3.3.4. Confocal Qualitative Observation of Cell Adhesion

**Figure 11:**
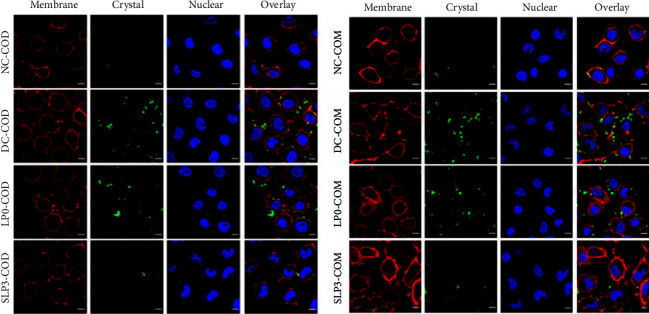
Confocal qualitative observation of cell adhesion. Green fluorescence is FITC-labeled nano-COM and COD crystals, red fluorescence is the cell membrane stained by DiI, and blue fluorescence is the cell nucleus stained by DAPI. Oxalate damage concentration: 2.6 mmol/L; damage time: 3.5 h; polysaccharide concentration: 80 *μ*g/mL; recovery time: 12 h nano-COM and nano-COD concentration: 200 *μ*g/mL; adhesion time: 1 h Scale bars: 10 *μ*m. NC: normal control, DC: damaged control.

The adhesion of nano-COM and nano-COD crystals on the cell surface before and after SLPs recovered damaged HK-2 cells was observed using confocal microscopy (Figure 11). For nano-COM crystals, the normal group had few cell adhesion crystals, whereas the damage group had the most adhesion crystals. After the damaged cells were recovered by LP0 and SLP3, the amount of adhesion crystals decreased, and the SLP3 recovery group had few adhesion crystals, close to the normal group. This result showed that SLPs had a recovery effect on the damaged cells, and SLP3 with high sulfate content had a good recovery effect. Nano-COD crystals had similar laws as nano-COM, but the adhesion amount of nano-COM crystals in the same group of cells was greater than that of nano-COD.

#### 3.3.5. Quantitative Detection of the Proportion of Adherent Crystal Cells

**Figure 12:**
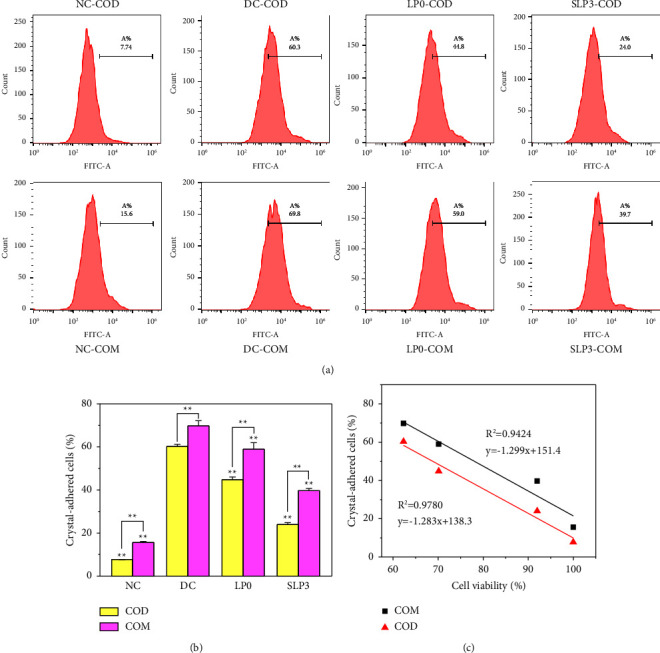
Quantitative detection of cell adhesion to nano-COM and nano-COD crystals before and after SLPs recovered damaged HK-2 cells. (a) The proportion of cells that adhere to the crystals was quantitatively detected by flow cytometry; (b) Statistical results of the proportion of cells that adhere to the crystals; (c) Relationship between cell viability and the adhesion amount of COM and COD crystals on the cell surface. Oxalate damage concentration: 2.6 mmol/L; damage time: 3.5 h; polysaccharide concentration: 80 *μ*g/mL; recovery time: 12 h nano-COM and nano-COD concentration: 200 *μ*g/mL; adhesion time: 1 h NC: normal control, DC: damaged control. Compared with the DC group, ^*∗*^*P* < 0.05, ^*∗∗*^*P* < 0.01.

Figure 12 shows the percentages of cell adhesion to nano-COM and nano-COD crystals before and after SLPs recovered damaged HK-2 cells. For nano-COD crystals, the percentages of cells that adhered to the crystals in the normal and damage groups were 7.74% and 60.3%, respectively, whereas the percentages of cells that adhered to the crystals in the LP0 and SLP3 recovery groups were 44.8% and 24.0%, respectively. By contrast, the percentages of cells that adhered to nano-COM crystals in the normal, damage, LP0, and SLP3 recovery groups were 15.6%, 69.8%, 59.0%, and 39.7%, respectively. It can be seen that there is a negative correlation between cell vitality and the adhesion amount of COM and COD crystals on the cell surface (Figure 12(c)), that is, the greater the cell vitality, the less the crystal adhesion amount.

The above results indicated that SLPs had a recovery effect on the damaged HK-2 cells, and the recovery effect of SLP3 was stronger than that of LP0. In addition, the adhesion of HK-2 cells to nano-COM crystals was higher than that to nano-COD crystals. For example, the percentages of HK-2 cells in the normal group that adhered to nano-COM and nano-COD were 15.6% and 7.74%, respectively, whereas those in the damage group were 69.8% and 60.3%, respectively.

## 4. Discussion

### 4.1. Influence of the Sulfate Content in SLPs on Biological Activity

The biological activities of polysaccharides depend on molecular structure, including monosaccharide composition, glycosidic bond of the main chain, degree of substitution, degree of branching, sugar component, and conformation of the main chain [30], especially the type and the content of functional groups contained in polysaccharides. Some natural polysaccharides do not have biological activity or have weak biological activity, but their biological activity could be enhanced by chemical modification [31]. The sulfation modification of polysaccharides is a common method to improve the biological activity of polysaccharides. After the sulfate group is added to the sugar base, the biological activity of polysaccharides often increases [32].

Liu et al. [33] have modified *Codonopsis pilosula* polysaccharide (CP) through sulfation to obtain sulfated CP and shown that the antioxidative activities in vitro and in vivo and the hepatoprotective activities sulfated CP are stronger than those of CP. Wei et al. [34] have modified *Radix hedysari* polysaccharide (RHP) by sulfation and obtained four sulfated derivatives (RHPSs) with different degrees of substitution. Compared with RHP, RHPSs showed significant antitumor activities on A549 and BGC-823 cells in vitro. Wang et al. [35] have modified CPP by sulfation and obtained the sulfated derivative S–CPP. This derivative could attenuate inflammatory mediators by inhibiting the phagocytosis of macrophage cells, the production of NO, and the release of interleukin 6 (IL-6) and IL-1*β*, thereby enhancing the anti-inflammatory ability of polysaccharides. Bhadja et al. [36] have studied and compared the recovery effects of six seaweed polysaccharides with different sulfate contents (SPSs) on oxalate-damaged HK-2 cells. They have shown that the recovery ability of SPSs in the range of 0.1–100 *μ*g/mL was positively correlated with its sulfate content.

This study showed that four SLPs with different sulfate contents had recovery effects on the HK-2 cells damaged by oxalate, and the recovery effect was positively correlated with the sulfate content of polysaccharides. As the sulfate content of polysaccharides increased, the cell viability gradually improved (Figure 2), the cell morphology gradually recovered to near normal (Figure 3), and the healing ability gradually was restored (Figure 4) after the recovery of damaged HK-2 cells by SLPs. Because the repaired cells can effectively reduce crystal adhesion and crystal aggregation, that is, promote the excretion of urinary microcrystallites, it is conducive to inhibiting the formation of kidney stones.

### 4.2. Recovery Effect of SLPs on Damaged HK-2 Cells

HK-2 cells produce ROS when exposed to oxalate. ROS includes superoxide free radicals, hydroxyl free radicals, and hydrogen peroxide. The free radicals generated after an increase in ROS levels could cause oxidative damage to DNA, proteins, and lipids [37, 38]. SPS, which is a natural antioxidant, could effectively prevent the damage caused by oxidative stress and has minimal side effects on the human body [39]. Raguraman et al. [40] have isolated sulfated polysaccharides (SPs) from *Sargassum tenerrimum*, and these SPs showed the scavenging effect on the ROS induced by hydrogen peroxide in zebrafish. After SP protection was implemented at concentrations of 50, 100, and 150 *μ*g/mL for 1 h, ROS levels remarkably reduced from 342.6% in the hydrogen peroxide damage group to 264.3%, 260.1%, and 114.8%, respectively. Souza et al. [41] have found that the SP extracted from the red seaweed *Gracilaria birdiae* has antioxidant activity and could effectively scavenge hydroxyl and DPPH free radicals. In the present study, all SLPs could reduce the level of ROS (Figure 5), and a high sulfate content of polysaccharides resulted in decreased ROS levels and oxidative damage to cells.

The mitochondrial function is a key indicator of cell health, and it could be assessed by monitoring the changes in ΔΨ_*m*_ [42]. The decrease in ΔΨ_*m*_ is an early manifestation of apoptosis. Once ΔΨ_*m*_ is lost, cells enter irreversible apoptosis [29]. The ΔΨ_*m*_ of normal cells is higher than that of damaged cells, and oxalate induces cell mitochondrial dysfunction and destroys the redox homeostasis [43], thereby reducing ΔΨ_*m*_. Williams et al. [44] have found that compared with that in healthy people, the mitochondrial function of monocytes in CaOx stone formers is reduced. Polysaccharides can recover damaged cells and increase the cell ΔΨ_*m*_. Sun et al. [45] have found that green tea polysaccharides can recover oxidatively damaged HK-2 cells. After restore by green tea polysaccharides, the cell morphology of the damaged HK-2 cells gradually recovered, ROS decreased, and ΔΨ_*m*_ increased. The present study showed that SLPs could increase the ΔΨ_*m*_ of damaged HK-2 cells, and the increase in ΔΨ_*m*_ was positively correlated with the sulfate content of polysaccharides (Figure 6).

PS is one of the important phospholipid components of the cell plasma membrane and has important biological functions. Under normal physiological conditions, PS is usually concentrated in the lobules in the plasma membrane of the cell. However, under apoptosis and some special pathological conditions, the intracellular ATP supply is insufficient, and the cytoplasmic Ca^2+^ concentration increases. PS exists in the outer lobules of the plasma membrane, i.e., PS eversion [46]. The present study showed that cell damage could cause PS eversion on the cell membrane, and the PS eversion ratio of the damaged cells decreased after polysaccharide recovery (Figure 7(b)). The amount of PS eversion on the cell surface was negatively correlated with cell viability (Figure 7(c)).

### 4.3. Inhibition of Cell Adhesion to CaOx Crystals after SLP Recovery

The adhesion of CaOx crystals to HK-2 cells is the key to the formation of kidney stones [9]. Certain proteins on the surface of HK-2 cells, such as ANXA1 and CD44, could promote the adhesion of CaOx crystals [8–10]. The ANXA1 is a protein with a molecular weight of 38 kDa, and it can bind to cellular membranes in a Ca^2+^-dependent manner [47]. Its function is related to the regulation of membrane transport and cell adhesion [48]. CD44 is a transmembrane glycoprotein, and its main ligand is hyaluronan. The combination of the two could activate various signal transduction pathways, leading to cell proliferation, adhesion, and migration [49]. The present study showed that after the oxalate-induced HK-2 cell damage, the expression levels of ANXA1 and CD44 proteins are increased, and the adhesion of CaOx crystals on the cell surface is enhanced, thereby increasing the risk of kidney stone formation. However, after the damaged cells were recovered by SLPs, the expression levels of the two proteins decreased, and the expression level of ANXA1, especially CD44, was approximately negatively correlated with cell viability (Figures 8(e and f)), indicating that SLPs had a recovery effect on the damaged HK-2 cells.

Studies have shown that the growth process of crystals is slow. The transit time for crystals to cross the kidney is 5–10 min, and crystals cannot grow large enough to block renal tubules during this time [50]. Therefore, nonadherent crystals do not pose a big risk to the formation of kidney stones. Only the crystals adhered to HK-2 cells could have sufficient time to continue to grow on the cell surface or gather mutually to form large crystals, eventually leading to the formation of kidney stones [51]. Studies have also shown that cell damage aggravates the adhesion of crystals, and polysaccharides could effectively reduce the adhesion of crystals after recovering damaged cells [39]. Zhang et al. [52] have found that the adhesion of nano-COM crystals is closely related to cell state, and damaged HK-2 cells adhere to more crystals than normal cells. The cells recovered by *Porphyra yezoensis* polysaccharides could inhibit the adhesion of COM crystals and promote the endocytosis of the adhered crystals. Both effects reduced the adhesion of crystals on the cell surface. The present study showed that the adhesion of CaOx crystals on the cell surface decreased after the recovery of damaged HK-2 cells by SLPs, and the amount of crystal adhesion was negatively correlated with the sulfate content of polysaccharides (Figures 10–12). The amount of crystal adhesion on the cell surface after recovery with the high sulfate content of SLP3 was less than that of LP0.

**Figure 13:**
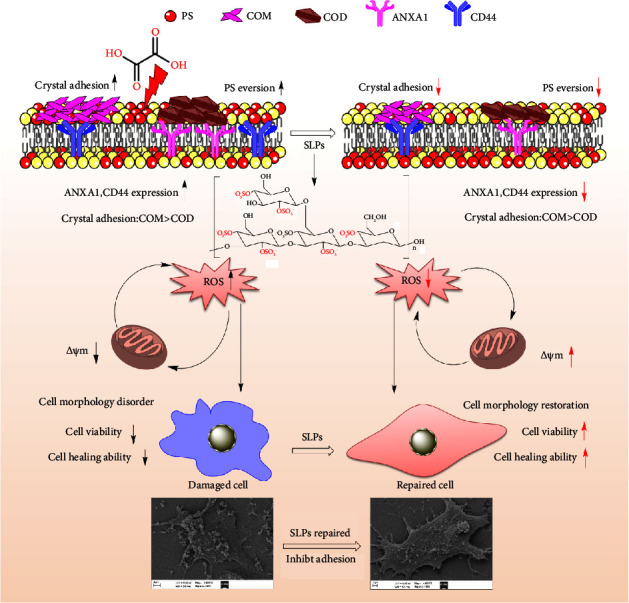
Model diagram of SLPs recovered damaged HK-2 cells and inhibited adhesion of nano-CaOx crystals.

In addition, this study showed that the toxicity of nano-COM crystals was greater than that of nano-COD crystals, and the toxicity of the adhesion of HK-2 cells to nano-COM crystals was remarkably stronger than that to nano-COD crystals. Considering that COM damages cells more than COD [53], reducing COM adhesion was found to be conducive to inhibiting the formation of CaOx kidney stones (Figure 13).

## 5. Conclusions

Four kinds of LP (i.e., LP0, SLP1, SLP2, and SLP3) with sulfate contents of 0.73%, 15.1%, 22.8%, and 31.3%, respectively, could effectively restore the oxidative damage of HK-2 cells induced by oxalate. Compared with the damage group, the SLPs recover groups have increased cell viability; reduced ROS production, ΔΨ_*m*_ loss, and PS eversion ratio; enhanced cell migration ability; and reduced expression levels of ANXA1, CD44, and HSP90 on the cell surface. SLPs could inhibit the adhesion of CaOx crystals by recovering damaged HK-2 cells, and the adhesion ability of the cells to COD crystals is weaker than that to COM crystals. The biological activity of polysaccharides is positively correlated with their sulfate content. Thus, SLP3 with the largest sulfate content has the best recovery effect on damaged cells and the strongest ability to inhibit the adhesion of nano-CaOx. This helps to inhibit the formation of kidney stones. This finding implied that SLPs, especially SLP3, may be used as green drugs to prevent and treat kidney stone formation.

## Figures and Tables

**Table 1:** Cell migration rate within 24 h after recovery HK-2 cells by SLPs.

SLPs	NC	DC	LP0	SLP1	SLP2	SLP3
–OSO_3_^–^ content (%)			0.73%	15.1%	22.8%	31.3%
L (*μ*m)	852.1 ± 21.8	638.7 ± 24.1	663.7 ± 14.2	686.1 ± 24.3	794.0 ± 5.8	829.4 ± 21.2
*v* (*μ*m·h^−1^)	35.5 ± 2.4	26.6 ± 1.6	27.6 ± 2.6	28.6 ± 1.0	33.1 ± 1.2	34.6 ± 1.82

## Data Availability

The data used to support the findings of this study are available from the corresponding author upon request.
